# Use of whole exome sequencing for identification of genetic variants related to Growth Hormone Deficiency and Short Stature: A Family-Based Study

**DOI:** 10.12669/pjms.39.5.7601

**Published:** 2023

**Authors:** Shatha Alharazy, Muhammad Imran Naseer

**Affiliations:** 1 Shatha Alharazy Department of Physiology, Faculty of Medicine, Center of Excellence in Genomic Medicine Research, King Abdulaziz University, Jeddah 21589, Saudi Arabia; 2 Muhammad Imran Naseer Department of Medical Laboratory Technology, Faculty of Applied Medical Sciences, Center of Excellence in Genomic Medicine Research, King Abdulaziz University, Jeddah 21589, Saudi Arabia

**Keywords:** Whole exome sequencing, Growth hormone deficiency, Vitamin-D, Vitamin-D deficiency, Polymorphisms, Short stature

## Abstract

**Objective:**

Genetic polymorphisms in genes involved in growth process and Vitamin-D metabolism form a significant etiology behind growth hormone deficiency and short stature. The aim of this study was to explore for known and unknown genes and variants related to growth hormone and short stature in a family based study using whole exome sequencing (WES).

**Method:**

This family-based study included a family with members diagnosed with growth hormone deficiency, short stature and Vitamin-D deficiency (four boys affected and four boys non-affected). The participants were recruited from King Abdulaziz University Hospital (Jeddah, Saudi Arabia) and referred to King Fahad Centre for Medical Research (Jeddah, Saudi Arabia from April 2022 to June 2022. The consanguineous parents and one of the affected boys (aged 16 years old) underwent WES.

**Results:**

Several variants in RNPC3, ACAN, GC, VDR and LRP2 were identified in index cases but not in controls. Novel frameshift and splice region variants in *RNPC3* (c.358dupA, p.Arg120fs) were detected. Other missense variants were also observed including variants in *ACAN* (c.2591C>T, c.2789G>T, c.2815T>A, c.4207A>G, c.4523A>C and c.7119C>G), *GC* (rs4588 and rs7041) *and LRP2* (rs2075252 and rs1991517). A start loss variant in *VDR* (rs2228570) with high impact was also observed.

**Conclusions:**

Our findings suggest a potential association of these variants with growth hormone deficiency and short stature. In this study, novel pathogenic variants in RNPC3 were revealed as well as other variants in ACAN and in genes related to Vitamin-D metabolism (GC, VDR and LRP2) that some or all might be associated with growth hormone deficiency. Further large-scale studies are required to address the association of these variants with growth hormone deficiency and its subsequent short stature.

## INTRODUCTION

Genetic polymorphisms in genes involved in growth process form a significant etiology behind GH deficiency (the isolated form) and short stature. It has been observed by a previous study that isolated GH deficiency can be caused by genetic defects in 10% of subjects approximately with a greater prevalence of 34% in familial type in comparison with sporadic cases which showed a prevalence of 4%.^1^ The incidence of short stature linked with isolated GH deficiency ranges between one in 4000 and one in 10,000 live childbirths.^2^ Around five to 30% of isolated GH deficiency cases are considered familial as a result of genetic variations in genes related to metabolic pathway of GH, mainly GH gene and GH releasing hormone receptor gene.^3^

Several factors can influence GH secretion and thus short stature including nutritional, lifestyle, hormonal and genetic factors.^4^,^5^,^6^ Among the important nutritional factors, Vitamin-D is considered to be vital for bone growth and mineralization by its role in regulating calcium and phosphate metabolism.^2^ A large number of studies have been investigating the influence of Vitamin-D on human health and adverse outcomes of Vitamin-D deficiency. Severe Vitamin-D deficiency in pediatric age causes rickets which is characterized by delayed growth in stature and bone malformation.^7^

The interaction between Vitamin-D and the GH or insulin-like growth factor is too complicated and it is not completely interpreted up to date. GH regulates the activity of one α-hydroxylase, the enzyme responsible for the second activation step of 25-hydroxyVitamin-D (25(OH) D) in the kidney. In addition, the effect of GH in moderating Vitamin-D metabolic pathway might be facilitated by insulin-like growth factor one.^8^ Several studies have reported an association between genetic polymorphisms in genes related to Vitamin-D (mainly Vitamin-D receptors) and human height.^9^,^10^ However, the association of many other genes contributing in Vitamin-D metabolism with GH deficiency and short stature has not been adequately addressed.

Genetic polymorphisms in genes involved in growth process form a significant etiology behind isolated GH deficiency as well as short stature.^11^ In addition, the recognition of the genetic defects (whether related to GH or Vitamin-D metabolic pathway) underlying the pathology of short stature and GH deficiency is vital for families in clinical practice. It terminates the doubt and saves unneeded investigations and managements and helps in proper genetic counseling, thus determining potential comorbidities associated with short stature.^12^ Genetic disorders or flaws can be detected efficiently by whole exome sequencing (WES) or next generation sequencing. WES is successful in screening of the whole coding region of DNA with less time and cost.^12^ With use of WES, we are expecting to identify part of the genetic etiology of GH deficiency through determination of novel genes or associated variants with GH deficiency and short stature. Therefore, the aim of this study was to investigate the association of known and unknown genes and variants involved in GH and Vitamin-D metabolism leading to GH deficiency and short stature accordingly in a family based study using WES.

## METHODS

### Study procedure and recruitment

A family-based study was conducted from April 2022 to June 2022 by including a family with members diagnosed with Vitamin-D deficiency (total 25(OH) D level below 20 ng/ml), GH deficiency (GH level below 7 µg/L) and short stature (below third percentile). The family was recruited from King Abdulaziz University Hospital (Jeddah, Saudi Arabia) and referred to King Fahad Centre for Medical Research (Jeddah, Saudi Arabia) after obtaining the ethical approval for conducting the study.

### Ethical Approval

The ethical approval of this study was taken from the Research Ethical Committee in Unit of Biomedical Ethics, Center of Excellence in Genomic Medicine Research (CEGMR), King Abdulaziz University (05-CEGMR-Bioeth-2018).

**Fig.1:**
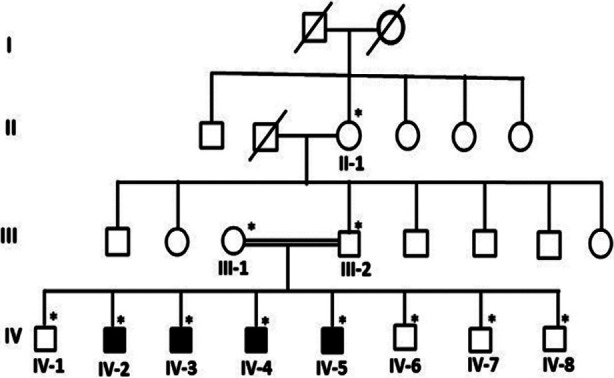
Showing the details of family members in the form of pedigree. All the affected members marked are black and the * represent the sample available for the study.

The sample collection and experimental work was following the international guidelines of Helsinki 2013 Declaration. Participants signed detailed consent for participation in this study. In minor participants, the parent of the child/minor signed the consent. Each participant was interviewed and full medical history was recorded. A pedigree for multiple generations of the participating family was documented as shown in Fig.1. Fasting blood samples were collected (in EDTA and red top tubes) from each participating subject for biochemical and genetic analysis.

### Blood biochemical analysis

Measurement of total serum 25(OH) D was done by chemiluminescence immunoassay, using a LIAISON auto-analyzer (DiaSorin Inc., Stillwater, MN, USA) while directly measured free 25 (OH) D was measured by immunoassay using ELISA kit (KAPF1991, Future Diagnostics Solutions B.V., Wijchen, Netherlands). Vitamin-D binding protein (VDBP) was assessed by quantitative sandwich enzyme immunoassay using Quantikine® ELISA (DVDBP0B, R&D Systems, Minneapolis, MN, USA). Quantification of albumin, calcium, phosphate, magnesium, lipid profile, blood glucose, renal and liver function in serum was done through the colorimetric method using a VITROS 250 Clinical Chemistry auto-analyzer (Ortho-Clinical Diagnostics Inc., Rochester, NY, USA).

### Genomic DNA extraction and quantification

Genomic DNA was extracted (from blood samples collected in EDTA tubes) through QIAamp genomic DNA extraction kit according to the manusfacturers’s protocols (https://www.qiagen.com/pk/products/top-sellers/qiaamp-dna-mini-kit/#orderinginformation). Genomic DNA quantification was performed with Nanodrop spectrophotometer (https://www.thermofisher.com/order/catalog/product/ND-LITE-PR) and visualized with CYBR Safe (Thermofisher, USA) dye via running on 1% agarose horizontal gel electrophoresis apparatus (https://www.cleaverscientific.com/electrophoresis/horizontal-gel-systems/). Samples with concentration of higher than 50 ng/µl were stored, and lower than the desired amount was re-extracted. Purified genomic DNA was stored at 4^o^C refrigerator.

### Whole exome sequencing (WES)

WES was performed for the samples of the two parents and for one of the affected boys (IV-3). Data generation and analysis involved several steps including data generation and data analysis.

### WES data generation:

### SureSelect-V7 Post exome library capture

This was the first step of capturing the coding regions of human genome. SureSelect-V7 post was a ready to use exome library capture kit designed by Agilent Technologies USA (https://www.agilent.com/en/promotions/sureselect-human-all-exon-v7). It contained “BAITS” probes of biotinylated single stranded RNA library for hybridization and capturing fragmented genomic DNA of sample in each library preparation process. There are several other exome library capture kits available in the market; however, SureSelect-V7 post has the advantage of capturing more than 125 plus reads lengths.

### Clustering and sequencing

Successful libraries were forwarded for clustering the sequence fragments and run through next generation sequencing platform (NovaSeq 6000 System, Illumina USA). Illumina platforms provide Flow cells for clustering the sequence reads through repeated synthesis of DNA fragments. Both strands of target DNA are synthesized according to template sequences captured previously in libraries. These completed clusters were then run on sequencer to obtain sequence raw data of the two. Successful runs followed extensive data analysis as written in the upcoming section.

### WES data analysis:

### Obtaining FASTQ files RAW data QC check

WES sequencing reads were obtained in FASTQ files format from Illumina machines. This data may contain some low signals which may lead to false positive results. To reduce the risk of false positivity, reads with Phred score below 20 were trimmed out and the rest will be allowed. It was performed by the latest bioinformatics Illumina packages called “bcl2fastq v2.20.0” (https://www.bioinformatics.babraham.ac.uk/projects/fastqc).

### FASTQ alignment

The sequences obtained in FASTQ passed from the above step were positioned against human reference genome which is termed as “alignment”. This alignment was achieved through Borrow-Wheel alignment tool (http://bio-bwa.sourceforge.net/bwa.shtml) and human reference genome hg19 (http://hgdownload.cse.ucsc.edu/goldenPath) with converting FASTQ raw data files to BAM files format.

### Duplicate reads deletion

Some of the sequences may appear redundant or repeated or duplicate in the BAM files. Therefore, they were further trimmed to single reads through “Picard-tool”. This was done through open source tool of Broad Institute of United Kingdom (http://broadinstitute.github.io/picard/).

### Variants annotations and variant call files generation

The obtained BAM files in the preceding step were then annotated by Genome Analysis Toolkit (http://www.broadinstitute.org/gatk). It is an outstanding and effective tool for variant detection in next generation sequencing data. Single nucleotide polymorphisms (SNPs) or variants at this phase were identified at nucleotide resolution. SNPs recognized were contrasted with genomAD databases (https://gnomad.broadinstitute.org/), SnpEff (http://snpeff.sourceforge.net/SnpEff.html) and 1000 genome (https://www.internationalgenome.org/).

### Variants prioritization

For variant prioritization, the coding and splicing regions of genes related to Vitamin-D metabolism, GH and short stature were evaluated using available online database for these variants (http://www.1000genomes.org, http://www.ncbi.nlm.nih.gov/SNP/ and http://gnomad.broadinstitute.org). Exonic and splicing junction indels and variants in genes causing protein level changes were targeted. Synonymous and intronic variants were excluded as well as variants in untranslated and intergenic regions. Candidate genes were reviewed in PubMed publications and the Online Mendelian Inheritance in humans’ database.

The obtained variants were filtered by applying various criteria including frequency, genomic position, protein effect, quality and involvement with Vitamin-D metabolism, GH deficiency and short stature. Screening for variants in main genes related to GH deficiency, short stature and Vitamin-D metabolism was performed. These genes included GH1, GHR, GHRHR, GHSR, HESX1, IGSF1, NPR2, PIK3R1, POC1A, SHOX, SLC5A5, THRB, TSHR, ACAN, RNPC3, BMP2, BTK, CYP2R1, DHCR7, CASR, MC1R, VDR, GC, CYP24A1, MC1R, LRP2, RXRA, CYP27B1 and CUBN. Following variants filtration, the number of variants in total decreased to 18 to 28 variants per sample.

The variants selected in this study after filtration were in the following target genes: RNA binding region containing 3 (RNPC3), Aggrecan (ACAN), Growth hormone receptor (GHR), Thyroid stimulating hormone receptor (TSHR), group-specific component (GC), Vitamin-D receptor (VDR) and low-density lipoprotein-2 (LRP2). Finally, the obtained results of WES analysis were contrasted with the results of our internal twenty-five controls. Matching of index samples with control samples was done for age, BMI, sunlight exposure, skin-tone and oral Vitamin-D intake. Controls were Vitamin-D sufficient without short stature, any skeletal deformities or any pathological abnormalities including endocrinal diseases.

## RESULTS

### General characteristics of the family participants

In our study, the results in Table-I show the characteristics of the participating subjects including their age, anthropometric measures, general data and biochemical analysis results. Levels of serum total 25(OH) D were deficient [25(OH) D <20 ng/ml] in both parents and in affected individuals who were previously diagnosed with GH deficiency, hypothyroidism and short stature (height<3rd percentile). In comparison, non-affected individuals showed sufficient serum 25(OH) D and were free from any illness.

**Table-I:** General and biochemical characteristics of the participating family.

Variables	Family participants									
	Parents		Affected children				Non-affected children			
	III-1	III-2	IV-2	IV-3	IV-4	IV-5	IV-1	IV-6	IV-7	IV-8
Age (years)	40	37	18	16	14	13	21	10	8	5
Sex	Male	Female	Male							
*Ethnicity*	*Asian (Pakistani)*									
Weight (kg)	90	57	42	33	34	30	64	25	20	13
Height (cm)	169	157	160	156	147	140	170	133	126	99
BMI (kg/m²)	31.5	23.1	14.5	13.6	14.2	14.7	25.3	15.7		
Hypertension/Diabetes*	No									
SBP/DBP (mmHg)	131/95	126/90	74/105	64/101	72/98	70/104	79/144	60/110	66/109	72/111
*Education*	*Illiterate*									
*Occupation*	*Private employee*	*Housewife*	-	-	-	-	-	-	-	-
*Skin tone (Fitzpatrick)†*	*Type III (medium white to olive)*	*Type III (medium white to olive)*	*Type IV (olive, mid brown)*							
Sun exposure (hr/week)	> 4	1-2	> 4	1-2	1-2	1-2	3-4	1-2	< 1	< 1
Use of sunscreen	No									
Dietary Vitamin-D intake (IU/day)	181	56	72	126	65	122	86	122	89	92
Physical activity	No	Yes	Yes	No	No	No	Yes	Yes	Yes	No
Smoking	No									
Serum total 25(OH)D (ng/ml)	9	17	15	12	16	15	33	37	42	46
Serum free 25(OH)D (pg/ml)	3.09	4.50	8.64	9.57	7.80	7.92	4.23	6.61	8.70	10.12
Serum VDBP (µg/ml)	508	418	741	450	333	520	508	689	761	606
Serum Albumin (g/L)	52	47	45	48	46	45	51	44	46	46
Serum Ca (mmol/L)	2.86	2.69	2.41	2.74	2.60	2.63	2.78	2.60	2.74	2.64
Serum PO_4_ (mmol/L)	1.39	1.48	1.69	2.11	1.80	1.63	1.69	1.81	1.93	2.04
Serum Mg (mmol/L)	0.90	0.92	0.90	0.90	0.80	0.80	0.90	0.70	0.80	0.80
Fasting blood glucose (mmol/L)	5.3	4.6	5.1	5.1	5.2	5.1	5.7	4.8	4.5	4.2
Serum AST (U/L)	38	19	24	36	30	32	40	33	36	38
Serum ALT (U/L)	59	18	22	40	26	30	38	32	29	28
Serum ALP (U/L)	116	63	146	185	207	181	120	249	279	235
Serum creatinine (µmol/L)	94	65	60	45	42	45	89	35	32	21
Serum total cholesterol (mmol/L)	5.1	5.5	2.7	3.7	3.1	2.4	3.1	3.3	3.6	3.7
Serum triglyceride (mmol/L)	2.35	2.38	0.49	0.46	0.50	0.35	0.17	0.75	0.50	0.72
Serum HDL-C (mmol/L)	1.2	1.6	1.2	1.6	1.4	1.2	1.6	1.5	1.4	1.3
Serum LDL-C (mmol/L)	2.77	2.80	1.28	1.86	1.46	1.05	1.41	1.52	1.97	2.08
Serum VLDL-C (mmol/L)	1.08	1.09	0.22	0.21	0.23	0.16	0.08	0.34	0.23	0.33

* According to medical records. BMI represents Body Mass Index; WC: Waist Circumference; HC: Hip Circumference; WHR: Waist Hip Ratio; SBP: Systolic Blood pressure; and DBP: Diastolic Blood Pressure †Fitzpatrick scale [20]. 25(OH)D is 25-hydroxyVitamin-D; VDBP is Vitamin-D binding protein; Ca is calcium; PO4 is phosphate; Mg is magnesium; AST is Aspartate Aminotransferase; ALT is Alanine Aminotransferase; ALP is Alkaline Phosphatase; HDL-C is high lipoprotein cholesterol; LDL-C is low density lipoprotein cholesterol; and VLDL-C is very low density lipoprotein cholesterol.

### Results of WES data

WES analysis showed several variants in genes associated with short stature, GH deficiency and hypothyroidism including ACAN, GHR, RNPC3 and TSHR (Table-II). Numerous variants were detected in ACAN (c.2591C>T, c.2789G>T, c.2815T>A, c.4207A>G, c.4523A>C, c.5293A>G, c.6235A>G and c.7119C>G), the gene encoding for aggrecan which is a main proteoglycan constituent of the growth plate extracellular matrix and articular cartilage. Additionally, a SNP (c.1651A>C) was identified in *GHR* (the gene encoding for GH receptor).

**Table-II:** Filtered WES results of genes variants related to GH deficiency, short stature and hypothyroidism.

Gene name	Cytogenic location	Gene accession number	HGVS.c	HGVS.p	Effect	Putative Impact	SNP	Disease association
*ACAN*	15q26.1	NM_013227.3	2591C>T	Pro864Leu	Missense variant	Moderate	rs3743398	Short stature
			2789G>T	Ser930Ile	Missense variant	Moderate	rs938608	
			2815T>A	Ser939Thr	Missense variant	Moderate	rs938609	
			4207A>G	Thr1403Ala	Missense variant	Moderate	rs12899191	
			4523A>C	Glu1508Ala	Missense variant	Moderate	rs2882676	
			5293A>G	Ile1765Val	Missense variant	Moderate	rs4932439	
			6235A>G	Ile2079Val	Missense variant	Moderate	rs1042630	
			7119C>G	Asp2373Glu	Missense variant	Moderate	rs3817428	
*GHR*	5q13-p12	NM_001242399.2	1651 A>C	Ile551Leu	Missense variant	Moderate	rs6180	(GH insensitivity syndrome, partial)
*RNPC3*	1p21.1	NM_017619.3	358dupA	Arg120fs	Frameshift variant & splice region variant	High	-	Isolated GH deficiency
*TSHR*	14q31.1	NM_000369.2	154C>A	Pro52Thr	Missense variant	Moderate	rs2234919	Hypothyroidism
		NM_001018036.2	742C>A	Arg248Ser	Missense variant	Moderate	rs3783941	
		NM_000369.2	2181G>C	Glu727Asp	Missense variant	Moderate	rs1991517	

HGVS.c/p stands for Human Genome Variation Society nomenclature for DNA reference sequence or protein reference sequence, respectively. SNP is single nucleotide polymorphism.

Furthermore, two pathogenic frameshift and splice region variants (c.358dupA, p.Arg120fs) with high impact were detected in *RNPC3* (a gene associated with isolated GH deficiency). Regarding *TSHR*, the gene encoding for thyroid stimulating hormone receptor, three SNPs were found including rs2234919 (c.154C>A), rs3783941 (c.742C>A) and rs1991517 (c.2181G>C). All found SNPS in *ACAN, GHR, RNPC3 and TSHR* were observed in index samples but not in controls except two SNPs in *ACAN* (rs4932439: c.5293A>G and rs1042630: c.6235A>G) and two other SNPs in TSHR (rs3783941: c.742C>A and rs1991517: c.2181G>C) which were both detected in index and control samples.

Other missense variants in genes involved in Vitamin-D metabolism (*GC, VDR and LRP2* genes) were also detected. Three SNPs (rs9016, rs4588 and rs7041) were identified in GC, the gene encoding for the main carrier of Vitamin-D (VDBP). Rs9016 was observed in the control samples and index samples while both rs4588 and rs7041 were observed in index samples but not in control samples.

A start loss SNP (rs2228570) was identified in *VDR* gene (the gene encoding for Vitamin-D receptor) with high impact as shown in (Table-III). In addition, three variants were also detected in *LRP2* (rs4667591: c.12628A>C, rs2075252: c.12280A>G and rs1991517: c.8614G>A) which is a gene encoding for receptor proteins involved in kidney transport of Vitamin-D binding protein 25(OH)D complex. All these SNPs in *VDR* and *LRP2 were* observed in index cases but not in controls except on SNP in *LRP2 (*rs4667591)

**Table-III:** Filtered WES results of genes variants related to Vitamin-D metabolism.

Gene name	Cytogenic location	Gene accession number	HGVS.c	HGVS.p	Effect	Putative Impact	SNP	Present in controls
*GC*	4q13.3	NM_001204307.1	1391A>G	His464Arg	Missense variant	Moderate	rs9016	Yes
			1364C>A	Thr455Lys	Missense variant	Moderate	rs4588	No
			1353T>G	Asp451Glu	Missense variant	Moderate	rs7041	No
*VDR*	12q13.11	NM_000376.2	2T>C	Met1?	Start lost	High	rs2228570	No
*LRP2*	2q31.1	NM_004525.2	12628A>C	Ile4210Leu	Missense variant	Moderate	rs4667591	Yes
			12280A>G	Lys4094Glu	Missense variant	Moderate	rs2075252	No
			8614G>A	Ala2872Thr	Missense variant	Moderate	rs2228171	No

HGVS.c/p stands for Human Genome Variation Society nomenclature for DNA reference sequence or protein reference sequence, respectively. SNP is single nucleotide polymorphism.

## DISCUSSION

The interaction between GH and Vitamin-D has been reported in previous studies. Vitamin-D deficiency and GH deficiency can both affect human height leading to short stature-5. In this study, we found through WES a number of genetic polymorphisms related to Vitamin-D metabolism, GH deficiency and short stature in a family with history of Vitamin-D deficiency, GH deficiency and short stature. We identified six missense SNPs in *ACAN* gene in the 16 years old boy presented with short stature in this study but not in healthy controls. These SNPs might be associated with short stature as they can influence the extracellular matrix of growth plate and articular cartilage, thus affecting bone growth and resulting in stop of growth in early stage.^13^ Previous studies have linked several other mutations in *ACAN* with familial short stature.^14^ Whether our observed SNPs in *ACAN* can be one of the potential genetic causes or risk factors underlying short stature is still needed to be further investigated in future studies.

In this study, it was very striking and prominent that we found novel high impact frameshift and splice region pathogenic variants in *RNPC3* (c.358dupA, p.Arg120fs) that are most likely to be associated with GH deficiency condition observed in our family case. In support of our finding, there are number of studies reporting the association of other different biallelic variants in *RNPC3* with GH deficiency.^15^-^17^ The *RNPC3* gene is involved in the splicing process of the precursor mRNA, a vital stage in all eukaryotes gene expression. RNPC3 encodes for a minor spliceosome component (the U11/U12-65K protein) that participates in the splicing of precursor mRNA when the noncoding introns are detected and eliminated. Mainly, introns are excluded by the major U2-dependent spliceosome while a lesser percentage of introns is excluded by the minor U12-dependent spliceosome.

The minor U12 introns are composing around 0.35% of all introns in human and they exist in 700-800 human genes approximately.^18^ This explains the splicing flaw in minor introns that might occur in the genes involved in the growth process due to *RNPC3* variants and therefore leading to GH deficiency. Our finding highlights the role of RNPC3 protein and importance of minor spliceosome in the functioning of genes needed for the growth process. Beside the observed variants in *RNPC3*, we found another variant in TSHR (c.154C>A) that might be linked to hypothyroidism status seen in the affected members of the studied family (IV-2 to IV-5). Previous studies showed an association of this variant with thyroid diseases including congenital hypothyroidism and Graves’s disease (hyperthyroidism).^19^,^20^

In addition, hypothyroidism has been observed previously in studies linking GH deficiency with *RNPC3*.^15^,^16^ Whether *RNPC3* variants might be a possible genetic factor underlying hypothyroidism is still unknown and needs further investigation. GH deficiency manifested by short stature is a multifactorial disease in which nutritional factor including Vitamin-D status plays an important role.^21^ Both GH and Vitamin-D are essential for the skeletal growth by interacting with each other. Nevertheless, the mechanism of this interaction is not fully understood.^8^

A number of studies have shown that GH deficiency was accompanied by low Vitamin-D levels.^21^ When we investigated Vitamin-D related polymorphisms in our subjects presented with Vitamin-D deficiency, WES revealed the presence of rs7041 and rs4588, the two most common polymorphisms in *GC* (encoding VDBP). This was not surprising as these polymorphisms were extensively reported to be associated with low 25(OH)D levels.^22^,^23^ Whether these common polymorphisms in VDBP might influence GH level is still cannot be concluded.

In the current study, we found a start loss variant in *VDR* which is also not unexpected as SNPs in *VDR* were reported earlier to be associated with Vitamin-D status as well as human height.^9^,^10^ Specifically, the observed SNP in *VDR* (rs2228570) was found previously to be associated with Vitamin-D deficiency in a study conducted on Turkish Cypriots.^24^ It has been suggested in a former study that VDR might modify the expression of GH either directly or by interfering with other transcriptional factors.^25^ To confirm the association of the observed Vitamin-D SNP with GH deficiency and short stature, further large-scale studies are required.

Finally, we observed in this study two genetic variants (rs2075252 and rs2228171) in LRP2. The *LRP2* gene encodes megalin receptor protein that binds to VDBP 25(OH) D complex and participate in renal reabsorption of this complex.^26^ It has been suggested that genetic variants in LRP2 might affect 25 (OH) D level.^27^ Our detection of rs2075252 SNP in the participated family with Vitamin-D deficiency suggests that this SNP might be associated with deficient 25(OH) D level. It also adds more evidence to our recent published finding that reported this SNP in six affected families diagnosed with Vitamin-D deficiency.^28^ Regarding rs2228171, there is still no report in the literature associating this SNP with Vitamin-D deficiency (according to our knowledge). However, this SNP has been reported in a recent study suggesting its association with gestational weeks and risk of preterm birth.^29^ The relationship of LRP2 with Vitamin-D and GH deficiency remains an interesting area for additional exploration.

### Limitations of the study

One of the limitations of this study is the inability of WES to cover the whole genome and its recognition of several rare variants with unidentified significance. In addition, the large produced data from WES and the unclear likelihood of causal association can increase the chance of false positive results. Another limitation is the small sample size of the family members that underwent WES. Therefore, WES analysis of DNA samples for the rest of the children is planned in the future.

## CONCLUSION

In conclusion, our study discovered novel pathogenic variants in RNPC3, suggested strongly to be associated with GH deficiency. We also observed several variants in ACAN and in some other genes related to Vitamin-D metabolism (GC, VDR, LRP2) that are all required to be further addressed in the future in large, public genomic databases with application of statistical analysis to confirm their association with GH deficiency and its consequences including short stature.

### Authors’ Contributions:

**SA:** Contributed to the study design and execution, data analysis and manuscript drafting.

**MIN:** Contributed in study design, data analysis, writing editing and review of the final manuscript.
